# Effectiveness of a mobile phone application to increase access to sexual and reproductive health information, goods, and services among university students in Uganda: a randomized controlled trial

**DOI:** 10.1186/s40834-020-00134-5

**Published:** 2020-10-31

**Authors:** Elly Nuwamanya, Robinah Nalwanga, Afra Nuwasiima, Janet U. Babigumira, Francis T. Asiimwe, Joseph B. Babigumira, Vitalis P. Ngambouk

**Affiliations:** 1 GHE Consulting, P.O Box 27011, Kampala, Uganda; 2 Department of Community Medicine and Public Health, Sahlgrenska Academy, University of Gothenburg, P. O Box 414, 40530, Gothenburg, Sweden; 3 Global Medicines Program, Department of Global Health, University of Washington, P.O. Box 357630, Seattle, WA 98195 USA

**Keywords:** Mobile phone application, Mobile health, Sexual and reproductive health, University, Students

## Abstract

**Background:**

University students are one of the most vulnerable groups to sexual reproductive health [SRH] threats like sexually transmitted infections [STIs], unwanted pregnancies, and unsafe abortions and often have limited access to SRH information, goods, and services. This study assessed the effectiveness of using a mobile phone application (APP) to increase access to SRH information, goods, and services among university students in Uganda.

**Methods:**

Using data from a double-blinded randomized controlled trial, participants were randomly assigned to both the intervention (APP) and control (standard of care) arms. We executed descriptive analyses for baseline demographic characteristics by intervention, difference in difference (DID), and quantile regression analyses for both primary and secondary outcomes.

**Results:**

The median age of participants was 21 years of age, and the majority were female (over 60%), unemployed (over 85%) and Christian (90%). Over 50% were resident in off-campus hostels and in a relationship. Between baseline and end-line, there was a significant increase in SRH knowledge score (DID = 2, *P* < 0.001), contraceptive use (DID = 6.6%, P < 0.001), HIV Voluntary testing and counselling (DID = 17.2%, P < 0.001), STI diagnosis and treatment (DID = 12.9%, *P* < 0.001), and condom use at last sex (DID = 4%,*P* = 0.02) among students who used the APP. There was a significant 0.98 unit increase in knowledge score (adjusted coefficient = 0.98, *P* < 0.001), a significant 1.6-fold increase in odds of contraceptive use (adjusted coefficient = 1.6, *P* = 0.04), a significant 3.5-fold increase in HIV VCT (adjusted coefficient = 3.5, P < 0.001), and a significant 2-fold increase in odds of STI testing and treatment (adjusted coefficient = 1.9, P < 0.001) after adjusting for demographic characteristics among APP users compared to the control group.

**Conclusion:**

A mobile phone application increased sexual and reproductive health information (knowledge score), access to goods (contraceptives), and services (HIV voluntary testing and counseling and sexually transmitted infection diagnosis and management) among sexually active university students in Uganda. Further technical development, including the refinement of youth-friendly attributes, extending access to the app with other platforms besides android which was pilot tested, as well as further research into potential economic impact and paths to sustainability, is needed before the app is deployed to the general youth population in Uganda and other low-income settings.

**Trial registration:**

MUREC1/7 No. 07/05–18. Registered on June 29, 2018.

## Background

Many of the low-income countries of sub-Saharan Africa have predominantly young populations. Uganda is one such country with 78% of the general population below 30 years of age and adolescents aged 15–19 years, accounting for a quarter of the female population [1]. Given this age distribution, youth-friendly health services in Uganda and other low-income countries are a current and ongoing public policy imperative. One critical aspect of youth-friendly health services is in the area of sexual and reproductive health (SRH): young, often unmarried people are often excluded from SRH services (and research reporting) despite being sexually active [2]. This means that access to SRH information, goods, and services is a right that remains unrealized by the youth due to myriad factors, including economic hardship, social stigma, and community norms [3]. Consequently, youth access to SRH information, goods, and services is poor, and is reflected by high rates of teen pregnancy and pregnancy-related health problems, high unmet need for contraception, and low access to modern contraception [4, 5].

Nearly all low-income sub-Saharan African countries have also undergone a rapid increase in mobile and broadband internet coverage; the region is projected to have 1 billion sim connections and 500 million broadband connections by 2020 [6]. Additionally, there is an increasing penetration of technology hubs and internet developers, and an emerging tech-driven economy driven by young entrepreneurs [6]. In Uganda, there are 18 million unique mobile subscribers, well over half of the population has access to a mobile phone, network coverage is high (67%), and there have been significant increases in 4G and mobile internet connections [7].

The confluence of SRH needs for a predominantly young population and high and increasing access to mobile internet presents a unique opportunity for the development of youth-friendly, innovative digital health intervention (DHIs), particularly interventions in mobile health (mHealth). A review of mHealth projects in Uganda identified over 20 applications, including use for health worker and patient training, healthcare delivery, treatment and patient management support, and health facility improvement [8]. These interventions tended to use predominantly voice and text messaging or apply devices, such as tablet computers, directly at the point of use or service [8].

One area of potential growth in mHealth is in the use of mobile phone apps (MPAs) to increase access to health services in general and SRH information, goods, and services, in particular. DHIs, particularly mHealth, are associated with key youth-friendly attributes, including confidentiality, convenience, and entertainment/information value. The leap from text messaging-based mHealth to internet-based MPAs maintains the confidentiality and convenience attributes and magnifies the information and entertainment value of mHealth interventions. It also allows the development of multiple platforms and connections that expand their utility from information-only use to use for connecting to goods and services. This enables complex, multifaceted interventions with multiple potential avenues of impact and a wide variety of client or consumer choice. Additionally, entertainment value and appeal portend the ability to advertise, a key determinant of market success and potential sustainability at scale of a commercial MPA.

As part of a broader project conducted over an 18-month period, we developed and pilot-tested a MPA to increase access to SRH information, goods, and services in a population of university students in Kampala’s capital, Uganda [9]. A detailed description of the development of the MPA has been described elsewhere (Development of a mobile phone application for access to sexual and reproductive health information, goods, and services in a low-income setting, Forthcoming). After MPA-development and as part of the impact evaluation, we conducted a study of acceptability and utilization of the MPA in which we assessed the proportion of eligible participants that accepted the MPA by downloading it and utilization as the proportion of eligible participants that used the app to access SRH information, goods and services in a six-month follow-up period (Acceptability and utilization of a mobile phone application to increase access to sexual and reproductive health information, goods, and services by university students in Uganda, Forthcoming). Acceptability was high at 84, and 82% of participants that downloaded the app used it to access SRH information, goods, and services over a six-month period (Acceptability and utilization of a mobile phone application to increase access to sexual and reproductive health information, goods, and services by university students in Uganda, Forthcoming).

Given satisfactory acceptability and utilization estimates, we hypothesized that access to the MPA would lead to an increase in SRH knowledge and lead to an increase in access to SRH goods and services among university students.

## Methods

### Study design

The study was a randomized controlled trial in which access to SRH information, goods, and services using a MPA (MPA-SRH) was compared to the standard of care of access to SRH information, goods, and services (SOC-SRH). A protocol of the trial, which was a part of a broader app development and impact evaluation project, has been published previously [9]. The trial was approved by the Mbarara University of Science and Technology Ethical Review Committee and the Uganda National Council of Science and Technology and was registered at MUREC1/7 No. 07/05–18 on 29th June 2018.

### Participants

The trial included students of Kyambogo University (KYU), the second-largest university in Uganda, which is situated in the capital city, Kampala. Eligibility criteria for inclusion into the trial were: (1) age 18 to 30 years, (2) self-reported sexual activity in the last six months, (3) more than 12 months to graduation, (4) access to an internet-enabled Android smartphone, and (5) informed consent. Trial participants were recruited in KYU halls of residence and KYU-affiliated hostels. Data were collected by in-person interviews in students’ rooms.

### Intervention

In the intervention group, trial participants were granted access to a MPA to enable their access to SRH information, goods, and services over a period of six months. Details of the development and features of the app are described elsewhere (Development of a mobile phone application for access to sexual and reproductive health information, goods, and services in a low-income setting, Forthcoming). Briefly, the MPA was developed in partnership with Gershom Technologies Ltd. using the Android operating system and archived on the Google Play store. The MPA was designed to link the different goods and services providers: healthcare facilities which provided SRH goods and services; Beyonic Uganda (https://beyonic.com/), a payments company which managed payments and co-payments; SafeBoda (https://safeboda.com/ug/), a transport company which managed the transportation of ordered goods and app users to receive services; and GHE Consulting (https://www.gheuganda.com/) for coordination, management, and oversight.

The MPA included the following features: (1) sign-up and sign-in; (2) a user module for ordering SRH goods (sanitary pads, male condoms, contraceptives, pregnancy tests, and pain killers) and services (HIV voluntary testing and counseling (VTC), STI diagnosis and treatment (D&T), family planning counseling, and general SRH consultation); (3) an SRH information module (menstrual period tracker, frequently-asked questions (FAQs), SRH tips, and a live chat); (4) a payments module to enable provider payments by GHE Consulting, copays by clients, and payments for transportation; (5) a delivery module to enable clients to track shipments, set up pickups for in-facility visits, and set up pick up points for products; and (6) a security module for authentication and password protection. Embedded in the MPA was an advertising interface to test potential sustainability at the scale of in-MPA advertising. While the study provided a major subsidy for SRH goods and services as well as transportation, there was a modest co-pay that was paid by app users through a link between mobile money and the Beyonic payment system. The co-pay was designed to test the utility of client payments in potential future iterations of the MPA after the pilot period.

### Procedures

Intervention group participants received a text message with a link to download the MPA, including directions on the set up of a MPA account and instructions on the use of the MPA. Upon download of the MPA, participants could gain access to SRH information through the four in-app portals. Participants could order SRH goods and have them delivered to their rooms or to a designated spot for pick up. Participants could also connect with health service providers to book SRH services and connect with transport providers to organize transportation to and from healthcare facilities. Participants in the SOC-SRH group received no intervention, i.e., accessed SRH information, goods, and services as they did before the onset of the trial.

### Outcomes

There were four primary outcomes in the trial all reflecting changes from baseline to end-line (end of six months follow-up period): SRH knowledge score (SRH information), use of contraceptives (SRH goods), use of HIV VTC (SRH services), and use of STI D&T (SRH services). There were two secondary outcomes in the trial, both reflecting changes from baseline to end-line: use of condoms and use of alcohol during the last sexual encounter (both behavioral and attributable to SRH information).

### Sample size

The sample size was calculated to detect a change between baseline and end-line in use of modern contraceptive goods. The prevalence of modern contraceptive use among sexually active youth aged 18 to 30 years at the time of study planning was 30.2%. Using the formula for the use of the Z-test for two sample proportions in studies with behavioral components, we calculated a sample size of 435 participants per group in order to obtain 90% power to detect a 15% change in modern contraception use in this population at a two-sided significance level of 5%. The sample size calculation assumed no design effect (d = 1) and was adjusted for a 10% non-response rate to obtain a minimum sample size of 479 per group (a total of 958 participants).

### Randomization and masking

After recruitment and informed consent, participants were informed that there was a 50–50 chance that they would be randomly assigned to gain access to the MPA (MPA-SRH) or be a part of the control group (SOC-SRH). The participants’ telephone numbers and unique study identification numbers were given to the app developer, who was responsible for randomization. Participants were randomized 1:1 to MPA-SRH and SOC-SRH using computer-generated random numbers. The research team, including providers at health facilities, transport providers, and payment technicians, and participants were blind to the intervention group, but the app developer was not.

### Statistical analysis

Data were double-entered and cross-validated, and all analyses were conducted in Stata version 16 (College Station TX, USA). We compared outcomes between participants randomly assigned to MPA-SRH versus SOC-SRH, ignoring potential changes in access to SRH information, goods, and services in the SOC-SRH group that may have been triggered by the use of the app by participants in the MPA-SRH group.

SRH knowledge scores were summed from correct responses from 17 different attributes and ranged from 0 to 17 (each question was awarded 1 point). Contraceptive use, HIV VTC, STI D&T, condom use during last sexual encounter, and alcohol use during the last sexual encounter were binary variables. We estimated the difference in changes from baseline to end-line (with 95% confidence intervals (CIs) and *p*-values) using differences-in-differences. We used the Wilcoxon rank-sum test for the knowledge score and the Z-test for proportions.

Using quantile regression (knowledge score) and a logit link function (access to modern contraceptive use, HIV voluntary counseling and testing, and STI testing and treatment), we estimated a mean differences-in-differences effect, adjusting for demographic characteristics. Models were estimated using maximum likelihood.

## Results

**Fig. 1:**
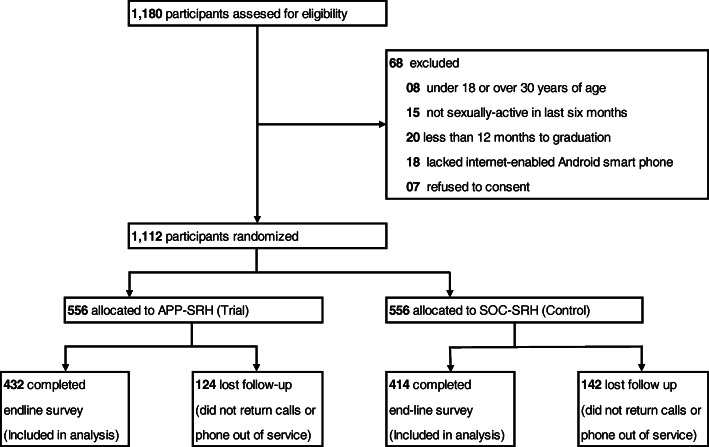
Trial profile

We enrolled participants from Oct 23rd, 2018, to 13th November 2018, and the last participant completed the six-month follow-up period on 25th May 2019. We enrolled (and assessed for eligibility) 1180 students and randomized 1112 students, 556 to the MPA-SRH (trial arm), and 556 to the SOC-SRH (control arm). Of the participants randomized, 846 (76.1%) completed the end-line survey, 432 (76.1%) in the APP-SRH arm, and 414 (74.5%) in the APP-SOC arm, constituting the sample for the analysis (Fig. 1).

The participants were predominantly women, resident in off-campus hostels, from urban hometowns, in relationships, in their first or second years of study, unemployed, and Christian. The loss to follow-up was predominantly due to participants not answering and returning calls or out-of-service telephone numbers. The baseline characteristics of the study participants are shown in Table 1. The median age of participants was 21 years of age, and the majority were female (over 60%), unemployed (over 85%) and Christian (90%). Over 50% were resident in off-campus hostels and in a relationship.

**Table 1:** Baseline characteristics of study participants by intervention

Characteristic	APP-SRH	SOC-SRH	All
	(*n* = 556)	(n = 556)	(*n* = 1112)
**Age at entry, years**	21 (2 [10, 11];)	21 (2 [10, 11];)	21 (2 [10, 11];)
**Sex**			
Male	214 (38.5)	193 (34.7)	407 (36.0)
Female	342 (61.5)	363 (65.3)	705 (63.4)
**Residence**			
Campus hall	137 (24.6)	133 (23.9)	207 (24.3)
Off-campus hostel	301 (54.1)	317 (57.0)	618 (55.6)
Rental home	104 (18.7)	86 (15.5)	190 (17.1)
Own home	02 (0.36)	03 (0.5)	05 (0.45)
Parent/ Guardian home	12 (2.16)	17 (3.1)	29 (2.6)
**Hometown**			
Urban	231 (41.6)	228 (41.0)	459 (41.3)
Peri-urban	184 (33.1)	189 (33.9)	373 (33.5)
Rural	141 (25.4)	139 (25.0)	280 (25.2)
**Marital status**			
Relationship	298 (53.6)	299 (53.8)	594 (53.7)
Single	239 (42.9)	241 (43.4)	480 (43.2)
Cohabiting	13 (2.3)	08 (1.4)	21 (1.89)
Married	04 (0.7)	08 (1.4)	12 (1.08)
Divorced	01 (0.2)	00 (0.0)	01 (0.09)
Widowed	01 (0.2)	00 (0.0)	01 (0.09)
**Year of study**			
First	174 (31.3)	195 (35.1)	369 (33.2)
Second	209 (37.6)	201 (36.2)	410 (36.9)
Third	147 (26.4)	134 (24.1)	281 (25.3)
Fourth	24 (4.3)	26 (4.68)	50 (4.5)
Fifth	02 (0.36)	00 (0.0)	02 (0.18)
**Employment**			
Unemployed	475 (85.4)	495 (89.0)	970 (87.2)
Employed	46 (8.3)	37 (6.7)	83 (7.5)
Volunteer	01 (0.18)	02 (0.4)	03 (0.27)
Self-employed	34 (6.1)	22 (3.9)	56 (5.04)
**Religion**			
Christian	500 (89.9)	486 (87.4)	986 (88.7)
Muslim	43 (7.7)	45 (8.1)	88 (7.9)
Others	13 (2.3)	25 (4.5)	38 (3.4)

Data are median (IQR; [range]), or n (%). APP = mobile phone application. SRH = sexual and reproductive health. SOC = standard of care.

Between baseline and end-line, there was a significant increase in SRH knowledge score, contraceptive use, HIV VTC, STI D &T, and condom use at last sex among APP users compared to the SOC-SRH (Table 2). There was a modest non-significant reduction in alcohol use at last sexual encounter among APP users compared to the SOC-SRH (Table 2). There was a significant 0.98 unit increase in knowledge score, a significant 1.6-fold increase in odds of contraceptive use, a significant 3.5-fold increase in HIV VCT, and a significant 2-fold increase in odds of STI testing and treatment after adjusting for demographic characteristics among APP users compared to the SOC-SRH (Table 3).

**Table 2:** Differences-in-differences analysis of primary and secondary outcomes

	MPA-SRH			SOC-SRH			DID [*P*-value: 95% CI]
	Baseline	End-line	Difference	Baseline	End-line	Difference	
Knowledge score	13 (11–15)	16 (15–16)	03 (z = −20.64, 0.02)	13 (11–14)	14 (13–15)	01 (z = − 9.01,0.01)	02 (z = 4.69,0.00)*
Contraceptive use	69.1%	82.2%	13.1%[0.00: 7.8–18.4]	73.7%	80.2%	06.5% [0.02:1.2–11.8]	06.6% [0.00:3.0–10.4]*
HIV VTC	68.0%	90.5%	22.5%[0.00:17.8–27.3]	64.2%	69.6%	05.4% [0.09: −0.6-11.3]	17.2% [0.00:13.0–21.4]*
STI D & T	52.3%	82.2%	29.8%[0.00:24.3–35.3]	52.3%	69.3%	17.0%[0.00:10.9–23.1]	12.9% [0.00:7.6–18.1]*
Condom use	66.2%	76.8%	10.4% [0.00: 5.1–16.3]	66.6%	73.0%	06.4% [0.03: 0.6–12.2]	04.0% [0.02:0.8–7.8]*
Alcohol use	7.6%	10.9%	03.3% [0.07: −0.3-7.0]	04.0%	07.5%	03.5%[0.02: 0.5–6.5]	− 00.2% [0.86:-.0.2–2.1]

*implies statistical significance at less than 5%

**Table 3:** Regression analyses

	Knowledge score		Contraceptive use		HIV VTC		STI testing and treatment	
	Unadj.	Adj.	Unadj.	Adj.	Unadj.	Adj.	Unadj.	Adj.
**Constant**	13.00 [0.00(12.73,13.26)]*	10.26 [0.00(7.63,12.69)]*	2.81 [0.00(2.32,3.39)]*	0.45 [0.41(0.06,3.11)]	1.79 [0.00(1.51,2.13)]*	0.33 [0.25(0.52,2.15)]	1.10 [0.27(0.03,1.30)]	0.27 [0.15(0.46,1.58)]
**Intervention**	0.00 [1.00(− 0.38,0.38)]	0.35 [0.07(− 0.03,0.734)]	0.81 [0.09(0.61,1.03)]	0.73 [0.02(0.56,0.96)]*	1.18 [0.18(0.92,1.52)]	1.55 [0.27(0.90,1.49)]	1.00 [1.00(0.79,1.27)]	1.02 [0.86(0.80,1.3)]
**End-line**	0.10 [−0.59,1.14)]	1.90 [0.00(1.42,2.37)]*	1.44 [0.02(1.06,1.96)]*	1.56 [0.01(1.1,2.22)]*	1.27 [0.08(0.97,1.67)]	1.44 [0.03(1.05,1.98)]*	2.16 [0.00(1.58,2.69)]*	2.48 [0.00(1.74,3.26)]*
Intervention * End-line	2.00 [0.29(1.42,2.58)]	0.98 [0.00(0.29,1.46)]*	1.43 [0.10(0.93,2.21)]	1.58 [0.04(1.02,2.46)]	3.53 [0.00(2.23,5.57)]*	3.57 [0.00(2.24–5.68)]*	2.04 [0.00(1.37,3.04)]*	1.99 [0.00(1.26,2.86)]*
**N**	1958	1958	1958	1954	1958	1946	1958	1954
**Log-likelihood**	–	–	− 1072.59	− 1043.67	− 1101.17	− 1077.2	− 1227.27	− 1181.64
**AIC**	–	–	2153.17	2147.33	2210.34	2212.41	2462.55	2423.27

* implies statistical significance at less than 5%: Coefficients and P-values and 95% confidence intervals in parenthesis

## Discussion

We assessed the effectiveness of a MPA to increase access to SRH information (measured as an increase in knowledge score and use of condoms and alcohol during the last sexual encounter), goods (measured as the proportion of participants using modern contraception), and services (a proportion that used HIV VCT and STI D&T) in a population of university students in Uganda. The MPA had a statistically significant benefit in increased SRH knowledge and increased access to SRH goods and services. MPAs are increasingly used in low- and middle-income countries for a wide range of applications, although applications in this setting are far exceeded by applications in high-income settings [12]. In sub-Saharan Africa and in the area of SRH, apps have been deployed to the public to support family planning [13] and to healthcare workers for improving provider quality of maternal and neonatal care [10, 14–19]. To our knowledge, this is the first report of an app deployed to members of the public to increase access to SRH information, goods, and services in the low-income setting.

Internet-enabled mobile phones and MPAs, including social media platforms, are popular among the youth around the world. In some settings, access to MPAs is associated with negative behavioral outcomes: as an example, a study in South Africa found increased odds of multiple sexual partners in the last year and higher prevalence of hazardous alcohol use with access to social media and messaging apps [20]. The configuration of MPAs to deliver health improving information is a potential avenue to counter the potential negative impact of other more generic apps such as social media and messaging apps on youth behavior while increasing access to health services such as SRH services. Our study demonstrated a significant increase in condom use during the last sexual encounter, suggesting a potential impact of a MPA on short-term behavioral change.

Research has established that the youth in low-income settings have significant SRH needs and face barriers to access to SRH goods and services, including physical access, legal and societal proscriptions, and societal barriers [11, 21]. There is substantial evidence that increasing youth-friendly information and access to SRH information and services are effective in fulfilling needs and alleviating barriers [11]. In our study, the MPA led to increased SRH knowledge and increased use of contraception, HIV VCT, and STI D&T, suggesting that the MPA was sufficiently youth-friendly to derive impact. Although there were some technical difficulties in the use of the MPA, these can be solved in future iterations of the MPA as plans are made for the transition to scale. And although our current focus was on a usable, deployable product, future prototypes will focus on refining youth-friendly attributes.

One weakness of the study was that the length of access to most components of the intervention (live chat and subsidies for goods, services, and information) were available for a short time (six months). As such, we are blind to the presence or absence of a sustained effect of increased knowledge or increase access to SRH goods and services on account of access to the MPA. Additionally, the trial was conducted among university students who are not generalizable to the general youth population in Uganda or other low-income countries. In fact, access to SRH information, goods, and services was high at baseline (13/17 knowledge score, over 70% access to contraception, 65% ever undergone HIV VCT, and 50% ever undergone STI diagnosis and treatment). This is compared to a general population contraceptive use prevalence of 29% among women of reproductive age [22] or 47% among unmarried women [1], and the prevalence of HIV testing among 15–19-year-olds of 44% in women and 53% in men [1]. University students are also different from the general population in their increased access to both smartphones and internet connections. Although the results of this study are not generalizable to the general youth population, we anticipate increasing access to smartphones and internet access [6, 23, 24] and plan to explore the feasibility and effectiveness of SRH MPAs in the general population in future studies.

The demonstration of the effectiveness of the MPA to increase access to SRH information, goods, and services, along with lessons learned in the pilot implementation and impact evaluation is a necessary precursor to the further development of the MPA with the goal of developing a sustainable product that can be rolled out to the general public. Before this goal is realized, further studies of the cost-effectiveness of the MPA and its potential economic impact at scale are needed, as are studies on the perception of potential advertisers, key partners in a possible future product, on the potential of the MPA to drive revenue.

## Conclusion

A mobile phone application increased sexual and reproductive health information (knowledge score), access to goods (contraceptives), and services (HIV voluntary testing and counseling and sexually transmitted infection diagnosis and management) among sexually active university students in Uganda. Further technical development, including the refinement of youth-friendly attributes, as well as further research into potential economic impact and paths to sustainability, is needed before the app is deployed to the general youth population in Uganda and other low-income settings. This will enable uniform access to the app by allowing users from other platforms other than the android platform, which was pilot tested.

## Data Availability

All the data used and presented in this study are available upon request.

## Abbreviations

APP: Mobile Phone Application
CIs: Confidence Intervals
D&T: Diagnosis and Treatment
DHIs: Digital Health Interventions
FAQs: Frequently Asked Questions
IQR: Inter Quartile Range
KYU: Kyambogo University
mHealth: Mobile Health
MPAs: Mobile Phone Apps
SOC: Standard of Care
SRH: Sexual and reproductive health
STI: Sexually Transmitted Infections
VTC: Voluntary Testing and Counseling
